# Graphene-Based Flexible Sensors for Simultaneous Detection of Ascorbic Acid, Dopamine, and Uric Acid

**DOI:** 10.3389/fbioe.2021.726071

**Published:** 2021-09-20

**Authors:** Shuaishuai Meng, Yaming Liu, Li Wang, Xixi Ji, Yun Chen, Tingting Zheng, Jie Yu, Huanhuan Feng

**Affiliations:** ^1^ Sauvage Laboratory for Smart Materials, Flexible Printed Electronic Technology Center, School of Materials Science and Engineering, Harbin Institute of Technology (Shenzhen), Shenzhen, China; ^2^ School of Materials Science and Engineering, Harbin Institute of Technology (Shenzhen), Shenzhen, China; ^3^ Peking University Shenzhen Hospital & Biomedical Research Institute, Shenzhen-PKU-HKUST Medical Center, Shenzhen, China

**Keywords:** 3D graphene, flexible sensing, simultaneous detecting, biological small molecules detection, biochemical detection

## Abstract

Many diseases are closely related to abnormal concentrations of ascorbic acid (AA), dopamine (DA), and uric acid (UA). Therefore, the detection of these small molecules is significant for monitoring life metabolism and healthy states. Electrochemical detection has been widely used to detect small molecules due to its good selectivity, high sensitivity, and good economics. Fabrication and application are two sides of the coin, and we cannot give up one for the other. Graphene (GN) is a very suitable material for electrochemical sensing due to its excellent catalytic performance and large specific surface area. It possesses many excellent properties but cannot hold itself alone due to its nanoscale thickness. Herein, we have fabricated three-dimensional (3D) GN nanosheets (GNSs) on flexible carbon cloth (CC) by thermal chemical vapor deposition (CVD). The GNSs/CC can successfully detect AA, DA, and UA simultaneously. We find that these GNSs/CC sensors show good performance with 7 h CVD modification. The linear ranges of AA, DA, and UA are 0.02–0.1, 0.0005–0.02, and 0.0005–0.02 mM, respectively. The detection sensitivity rates of AA, DA, and UA are 5,470, 60,500, and 64,000 μA mM^−1^ cm^−2^, respectively. Our GNSs/CC flexible sensors can be successfully applied in the human serum for UA detection. The result matches with commercial sensors very well.

## Introduction

Clinical medicine research has found that chronic diseases such as scurvy, Parkinson’s disease, and gout are highly correlated with abnormal concentrations of ascorbic acid (AA), dopamine (DA), and uric acid (UA) (Masliah et al., 2000; Sansuk et al., 2013; Du et al., 2014; Liao et al., 2014). The coexistence of AA, DA, and UA is very common in the extracellular fluid, serum, and central nervous system. They are important signals of the state of human physiological processes (Wightman et al., 1988; Tukimin et al., 2018). Therefore, the fabrication of selective sensors which can simultaneously detect these biological small molecules in real time is of substantial significance for the normal life activities of human beings and the early diagnosis of related diseases. Nowadays, there are various methods for detecting these small biological molecules: ultraviolet-visible spectroscopy (Han et al., 2014), fluorescence measurement (Hortigon-Vinagre et al., 2016; Tarabova et al., 2019), chemiluminescence (Nalewajko et al., 2004), gas chromatography–mass spectrometry (Xie, 2001; Lu et al., 2020), and high-performance liquid chromatography (Mesbah et al., 1989; Ianni et al., 2019; Qi et al., 2020). However, these methods require a long detection cycle and professional operation and are of high cost, which limit their efficient detection of these small biological molecules. As these three substances all contain special functional groups, they will be oxidized at a certain potential and exhibit electrochemical activity, which can be measured by current. Therefore, electrochemical methods can be used to monitor the concentrations of these small molecules (Hubbard et al., 2010). Electrochemical sensors have become research hotspots due to their low cost, short detection cycle, high sensitivity, and simple operation (Bakker and Telting-Diaz, 2002; Privett et al., 2010; Li et al., 2019; Karimi-Maleh et al., 2020). Zhu fabricated a novel electrochemical sensor based on carbon nanotube arrays for selective detection of DA or UA (Yang et al., 2019). Wu designed a heteroatom-doped carbon nanoparticle–ionic liquid composite as the electrochemical sensor for UA (Abbas et al., 2020).

The single detection of these small molecules using electrochemical sensors is quite mature, but there are relatively few reports of sensors involved in the simultaneous detection of these three small molecules. The main reason is that the oxidation potentials of these substances on the conventional bare electrodes overlap each other and often suffer from a pronounced fouling effect, which result in rather poor selectivity and reproducibility (Gao and Huang, 1998; Kumar et al., 2005; Premkumar and Khoo, 2005; Zare et al., 2005). In order to overcome the difficulty, new electrode materials must be developed to reduce the oxidation potentials of various substances and to improve the electrode properties. In addition, the sensing materials used in the sensors are mostly inorganic nonmetallic materials such as silicon, zinc oxide, or gallium arsenide. The single-crystal materials are rigid and brittle but not flexible, thus being limited in the fields of application such as irregular surfaces and large deformation (Kim et al., 2008; You et al., 2020). Graphene (GN) electrodes stand out among many modified materials due to their excellent conductivity, high ductility, high thermal conductivity, and large specific surface area (Stankovich et al., 2007; Wu and Drzal, 2012; Yang et al., 2017). Although the GN electrode possesses numerous excellent properties, it cannot hold itself alone due to its nanoscale thickness. Carbon cloth (CC) is a very good electronic substrate, which can integrate with flexible electronics and realize the sensing and detection of biochemical signals (Polovina et al., 1997; Ezzeddine et al., 2015; Hou et al., 2015).

Herein, we report a GNSs/CC flexible sensor using thermal chemical vapor deposition (CVD) to grow three-dimensional (3D) GN nanosheets (GNSs) on the CC substrate for the simultaneous detection of AA, DA, and UA. The GN is directly grown on the CC and firmly combined with the CC fiber and does not fall off easily compared with other physical adsorption techniques. The GNSs/CC sensor has combined the advantages of the flexibility of CC with the high chemical activity and large specific surface area of 3D GN. It possesses excellent catalytic activity for AA, DA, and UA and has the advantages of high sensitivity and a wide linear range. We found that the GNSs/CC sensors show good detection performance when the GN growth time is 7 h. The 7 h GNSs/CC electrodes have sufficient chemical active sites with high chemical reactivity, which can separate the redox peaks of these small molecules without forming dense GN layers to enter the diffusion control stage. In addition, the sensor has good anti-interference performance with regard to common ions and molecules in blood and has been successfully used to detect UA in the human serum, with a recovery rate of 104–124%. This work provides a simple and efficient tool for the simultaneous detection of multiple biochemical signals.

## Materials and Methods

### Fabrication of the GNSs/CC Flexible Sensor

Since carbon materials were hydrophobic, hydrophilic pretreatments were required prior to use. The specific operations were as follows: the samples were immersed in acetone solution and maintained at room temperature for 30 min to dissolve the organic matter on the surface of these samples. The samples were taken out of the acetone solution, then put into deionized water, and maintained for 30 min. The samples were removed from the deionized water, then put into 0.5 M sulfuric acid solution, and maintained for 30 min to oxidize the surface of the carbon materials, so that the oxygen-containing groups can be modified on the samples’ surface. The carbon materials were converted from hydrophobic to hydrophilic attribute, and then, they were naturally air-dried in air under room temperature for further detection.

**FIGURE 1:**
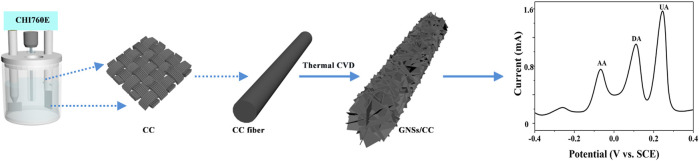
Schematic diagram of the fabrication process of the GNSs/CC sensor and the simultaneous detection of three small molecules (AA, DA, and UA).

To fabricate our GNSs/CC flexible sensor (Figure 1), we put a certain specification of CC into a tube furnace, introduced the protective gas argon (Ar) at a rate of 200 sccm, and heated the tube furnace to 1,100 °C to complete the carbonization of the CC fiber in Ar atmosphere. After some time, Ar was stopped and a mixture of hydrogen (H_2_) and carbon source (CH_4_) was introduced at a rate of 160 and 6 sccm, respectively, for 10 h (H_2_ was introduced to aid in the cleavage of CH_4_). On the basis of carbon fiber carbonization, GN with the 3D structure was generated *in situ* on the CC substrate by CH_4_ thermal decomposition reaction and the vertical growth of GN was completed. Finally, Ar was introduced to stabilize the GN and drain the mixture of H_2_ and CH_4_, and the tube furnace was cooled to room temperature to obtain the 10 h GNSs/CC electrode material.

In order to investigate the effect of the thickness of GN grown on CC on the electrochemical performance of the sensor, the GNSs/CC electrodes at different growth periods were fabricated by adjusting the introduction time of the carbon source to 0–10 h, which were 1 h GNSs/CC, 5 h GNSs/CC, 7 h GNSs/CC, and 10 h GNSs/CC electrodes, respectively. The fabricated GNSs/CC electrodes were quenched into 1 cm × 2 cm pieces with liquid nitrogen for further use. After use, they were washed repeatedly with anhydrous ethanol and ultrapure water to remove the residual substances on the surface to obtain good reproducibility.

### Basic Characterization of Materials

Scanning electron microscopy characterization: the cross sections of the samples were obtained by liquid nitrogen quenching. SEM using S-4700 was performed to observe their morphology, structure, and thickness under 15 kV and in the secondary electron mode.

Raman characterization: the samples fabricated in the experiment were characterized by Horiba microconfocal Raman spectroscopy. Selection of test parameters: the detection range was from 1,000 to 3,000 cm^−1^, the integration time was 3 min, the number of integrations was 20, and the selected laser wavelength was 625 nm. All tests were performed at room temperature.

BET: we carried out nitrogen adsorption and desorption experiments on GNSs/CC materials and calculated the specific surface area of GNSs/CC materials by measuring the saturation adsorption capacity of the nitrogen monolayer.

### Electrochemical Testing

Electrochemical impedance spectroscopy (EIS) is commonly used to examine the interface properties of modified electrodes, and potassium ferricyanide/potassium ferrocyanide is often used as an electron transfer redox probe (Pajkossy and Jurczakowski, 2017; Ciucci, 2019). Cyclic voltammetry (CV) scanning includes a pair of reversible redox peaks. In forward scanning, [Fe(CN)_6_]^4-^ loses electrons and becomes [Fe(CN)_6_]^3-^ through the oxidation reaction, while in reverse scanning, [Fe(CN)_6_]^3-^ gains electrons and becomes [Fe(CN)_6_]^4-^ through the reduction reaction. We measured the electrochemical impedance of various electrodes with 0.01 mM potassium ferricyanide/potassium ferricyanide solution at 0.1 Hz to 100 kHz with an amplitude of 0.05 V and a standing time of 2 s.

CV (Elgrishi et al., 2018) was used to investigate the electrochemical properties of GNSs/CC electrodes at different growth periods. The experiment was carried out on a CHI 760 E electrochemical workstation using the traditional three-electrode system, in which the GNSs/CC electrode was used as the working electrode, the counter electrode was a platinum plate, and the saturated calomel electrode (SCE) was used as the reference electrode. All potentials given were referred to SCE, and all electrochemical tests were performed at room temperature. 0.1 M phosphate-buffered saline (PBS) at pH 7.4 was used as the electrolyte solution for the performance test with a scan voltage of -0.6 to 0.4 V.

Differential pulse voltammetry (DPV) (Lin et al., 2006) was used to separately detect these small molecules (AA, DA, and UA), and the respective working linear range and sensitivity were obtained when the GNSs/CC electrode was used as the working electrode. Then, DPV was used to simultaneously detect these small molecules and obtain the working linear range and sensitivity of simultaneous detection.

### Detection in Real Blood Samples

In order to explore the performance and feasibility of our GNSs/CC sensor in the analysis of real blood samples, fresh human serum samples were analyzed with the sensor and the dry chemical analysis method was adopted by the hospital. Fresh blood samples were collected from Shenzhen Hospital of Peking University and analyzed within 1 hour after sampling. Collection of the samples was approved by the ethical committee of the hospital, and informed consent was obtained from each volunteer. This study was approved by the Medical Ethical Committee of Shenzhen Hospital of Peking University.

## Results and Discussion

### Characterization of Carbon Cloth and Graphene Nanosheets/Carbon Cloth

**FIGURE 2:**
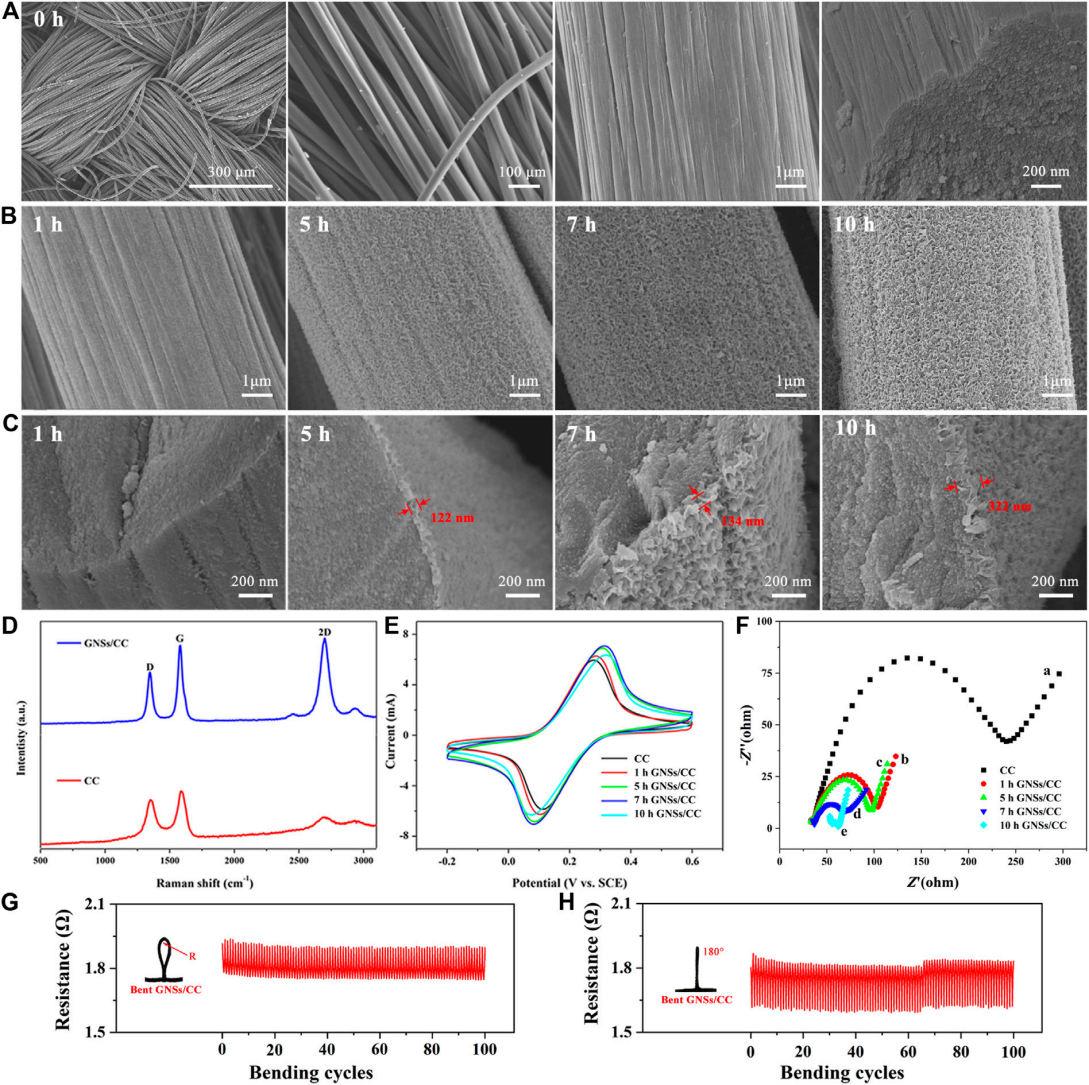
Relevant characterization of CC and GNSs/CC. **(A)** SEM of CC morphology and its cross section. **(B)** SEM of GNSs/CC with different growth periods of GN. **(C)** SEM of GNSs/CC cross sections with different growth periods. **(D)** Raman spectra of CC and GNSs/CC. **(E)** Cyclic voltammograms of CC and GNSs/CC. **(F)** EIS of different electrodes in 0.01 mM [Fe(CN)_6_]^3-/4-^ and 0.1 M KCl solutions. **(G)** Resistance of the GNSs/CC flexible sensor bent for 100 times at a radius R = 3 mm. **(H)** Resistance of the GNSs/CC flexible sensor bent for 100 times at 180°.

Figure 2A shows the SEM of CC. The surface of the bare CC fiber is relatively smooth, and the fiber is woven to form the CC. Figure 2B shows the SEM of GNSs/CC at different growth periods of GN. After growing GN on the CC for 1 hour, GN flakes appeared on the CC like shredded paper. The thickness of the GN layer increases with the growth time. As the growth time of GN increases, it can be seen that not only the thickness and density of GN increase significantly but also a 3D structure is gradually formed. This 3D network structure effectively increases its specific surface area and the contact area with small molecule substances, which provides more chemical active sites. It can be seen from the SEM of GNSs/CC cross sections (Figure 2C) that only after as long as 5 h of growth time can the full GN be grown and the thickness of GN coating on the outer layer of the CC fiber is 122 nm. With the increase in growth time, the density of GN and the diameter of the CC fiber gradually increase. The thickness of GN is 134 nm when it grows for 7 h. When the growth time is increased to 10 h, the GN on the CC touches each other, filling the space in the entire CC fiber and forming a unique porous 3D GN structure. At this point, the thickness of GN is around 320 nm. This special structure has a large specific surface area, which can further provide sites for the loaded active molecules.

We measure the specific surface area of GNSs/CC at different growth periods of GN. The specific surface area of the CC is not obtained, but after the growth of GN on the CC, the specific surface area increases significantly; the specific surface area of 1 h GNSs/CC is 1.3 cm^−2^ g^−1^. As the growth time of GN increases, its specific surface area gradually increases. This is because with the increase in growth time, the GN gradually becomes thicker and denser and forms a 3D structure. The special 3D structure increases the specific surface area of the composite material. Notably, the specific surface area of 5 h GNSs/CC is significantly increased (1.7 cm^−2^ g^−1^ at 5 h, 1.9 cm^−2^ g^−1^ at 7 h, and 2.0 cm^−2^ g^−1^ at 10 h). On the one hand, GN itself has a large specific surface area, and on the other hand, 5 h GNSs/CC forms a complete 3D structure. With the gradual formation of the 3D structure of GN, its specific surface area increases significantly, which is very consistent with the SEM images.

Raman spectra of CC and GNSs/CC show two characteristic peaks, which are the D peak at 1,346 cm^−1^ and G peak at 1,588 cm^−1^ (Figure 2D) (Sasaki et al., 2013). In the experiment, the *I*_D_/*I*_G_ ratios of CC and GNSs/CC are 0.8 and 0.7, respectively, indicating that the grown GN reduces the defects of the composite. Figure 2D shows that the 2D peak appears near 2,700 cm^−1^. Compared with CC, GNSs/CC has an obvious 2D peak, which can be used to judge that GN has been successfully fabricated. The peak strength ratio of *I*_G_/*I*_2D_ is used to assist in the analysis of the number of GN layers. In our work, the ratio of *I*_G_/*I*_2D_ is 0.88, which indicates that the grown GN has fewer layers. This result confirms the statement of 3D GNSs and indicates that the results measured by Raman spectroscopy are consistent with the results of SEM images.

We can judge whether the electrodes are modified and their conductivity is good or bad by comparing the peak currents on different modified electrodes (Zhang et al., 2019). Figure 2E shows the cyclic voltammograms of CC and GNSs/CC electrodes in 0.01 mM [Fe(CN)_6_]^3-/4-^ and 0.1 M KCl solutions. Compared with CC, the response current of GNSs/CC to [Fe(CN)_6_]^3-/4-^ increases obviously, and the larger the peak current, the larger the effective area of the electrode will be. Therefore, GN with the 3D structure fabricated by the thermal CVD method significantly increases the specific surface area of electrodes and provides more chemical active sites. In addition, it shows that the electron transfer ability of GNSs/CC electrodes is good and the excellent conductivity of GN plays an important role. Figure 2E also shows that the peak current gradually increases with an increase in the thickness of the GN layer and the maximum current value is obtained in 7 h GNSs/CC electrodes.

Figure 2F is the EIS of different electrodes. In the high-frequency part of EIS representing the resistance, CC electrodes present a large semicircle (curve a) with an impedance value of approximately 245 Ω. The impedance value decreases sharply to 82 Ω (curve b) when GN is modified on the CC fiber for 1 hour, indicating that the GNSs/CC electrodes have better electron transport capacity and electrochemical activity. Curves c, d, and e are the impedance curves of 5 h GNSs/CC, 7 h GNSs/CC, and 10 h GNSs/CC, respectively. The resistance values corresponding to the semicircles are 75, 47, and 11 Ω, respectively. With the prolongation in growth time of GN, the thickness of GN on CC increases gradually and the electron transfer resistance gradually decreases which indicates that the GNSs/CC electrodes can improve the transfer rate of electrons. Figures 2G,H show the resistance measurement of our sensor under different bending degrees. The results show that the resistance of the GNSs/CC flexible sensor remains stable during and after bending for 100 times, indicating that our sensor has the advantages of flexibility and performance stability. The reason is that CC is a very good electronic substrate with excellent flexibility, which can maintain its electrochemical performance under tough repeated bending.

### Cyclic Voltammetry for the Detection of Small Biological Molecules (Ascorbic Acid, Dopamine, and Uric Acid)

CV is used to investigate the catalytic performance of GNSs/CC electrodes in the detection of AA, DA, and UA. These small molecules are oxidized and lose electrons during the detection process, which generate electric current through electron transfer with the electrode (Ensafi et al., 2010; Zhou et al., 2014). The magnitude of the electric current is positively correlated with the concentrations of small molecules. Therefore, we can test the concentrations of a series of small molecules and their corresponding current values and find the linear range of these analytes.

**FIGURE 3:**
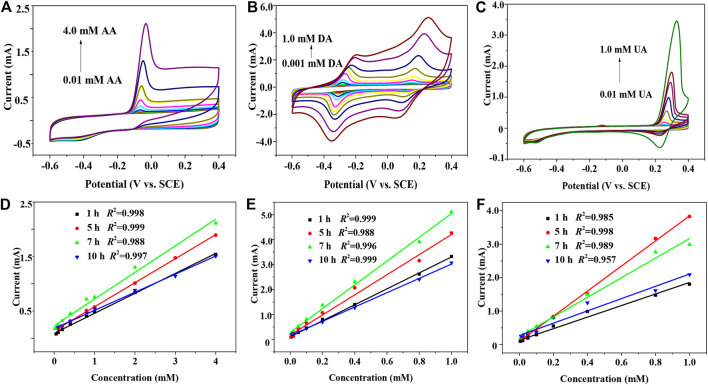
GNSs/CC electrodes with different GN growth periods are used to separately detect AA, DA, and UA by CV. **(A)** CV detecting AA alone. **(B)** CV detecting DA alone. **(C)** CV detecting UA alone. **(D)** Fitting results of the relationship between the current and concentration are obtained by detecting AA using GNSs/CC. **(E)** Fitting results of the relationship between the current and concentration are obtained by detecting DA using GNSs/CC. **(F)** Fitting results of the relationship between the current and concentration are obtained by detecting UA using GNSs/CC.

Figure 3A shows the cyclic voltammograms of 7 h GNSs/CC electrodes from -0.6 to 0.4 V at a rate of 50 mV s^−1^ in 0.01–4.0 mM AA and 0.1 M PBS solutions. The oxidation peak potential of AA on the electrode is −68 mV, and the oxidation peak potential and peak type of AA do not change much at different modified electrodes. In addition, the peak current of AA increases linearly with an increase in the AA concentration. Figure 3D shows the fitting results of the relationship between the current and concentration obtained by detecting AA with 1 h GNSs/CC, 5 h GNSs/CC, 7 h GNSs/CC, and 10 h GNSs/CC electrodes. The results show that the electrochemical response and sensitivity of GNSs/CC to AA are gradually enhanced with the increase in the thickness of GN. The maximum sensitivity of 244.3 μA mM^−1^ cm^−2^ and the minimum detectable concentration of 0.001 mM are obtained at 7 h GNSs/CC electrodes. The reason is that the presence of GN can improve the catalytic performance of AA when the growth time of GN is 1 h. Once the 3D structure of GN is formed, its ability to collect reaction electrons is further improved, and with the continuous improvement of this 3D structure, the active sites are further increased. The adsorption enrichment and catalytic capacity of small molecules reach the maximum when the growth time of GN is 7 h, so the response to AA is further improved and the sensitivity reaches the maximum. However, the detection sensitivity and response current of AA decrease when the growth time of GN increases to 10 h. It is possible that the GN coated on the surface of the CC is very thick; only the outer layer of GN participates in the reaction, while the inner layer of GN does not participate in the reaction, which becomes the invalid active site. Subsequently, we use our GNSs/CC electrodes to detect DA and UA by CV, respectively. Figures 3B,E show that the electrochemical response of GNSs/CC to DA gradually increases with an increase in the thickness of GN. The maximum sensitivity of 380 μA mM^−1^ cm^−2^ and the minimum detectable concentration of 0.0001 mM are obtained on the 7 h GNSs/CC electrodes. Figures 3C,F show that the electrochemical response of 5 h GNSs/CC electrodes to UA reaches the maximum. The sensitivity for detecting UA is 1872 μA mM^−1^ cm^−2^, and the minimum detectable concentration is 0.001 mM.

In order to verify the feasibility of our GNSs/CC electrodes for the simultaneous detection of three small biomolecules (AA, DA, and UA), we detect them simultaneously by CV using CC electrodes and GNSs/CC electrodes, respectively. In this experiment, the scan voltage range is −0.4–0.6 V, the scan rate is 0.5 mV s^−1^, and the scan time is 15 turns. An oxidation reaction will occur on the electrode when scanning occurs from the positive electrode to the negative electrode, and an oxidation peak will be obtained; when scanning occurs from the negative electrode to the positive electrode, a reduction reaction occurs, resulting in a reduction peak. The solution used is 0.1 M PBS solution with pH 7.4, and the concentrations of AA, DA, and UA target analytes are 0.8, 0.01, and 0.1 mM, respectively. Supplementary Figure S1 shows the cyclic voltammograms of different electrodes for the simultaneous detection of AA, DA, and UA. The results show that only two oxidation peaks of these three small molecules appear on the CC electrodes and the peak widths are larger, indicating that the oxidation peaks of the bare CC electrodes overlap each other in the detection of these substances. Therefore, the CC electrodes cannot be used to detect AA, DA, and UA simultaneously. On the contrary, the oxidation peaks of these three small molecules are obviously displayed on the GNSs/CC electrodes, the position and shape of the oxidation peaks are changed, and the width of the peaks is narrow. The three oxidation peaks that are initially close to each other are negatively shifted to different degrees, which realizes the separation of the three small molecular oxidation peaks. Supplementary Figure S1 also shows that the oxidation peak potentials of AA, DA, and UA is at −76, 190, and 327 mV, respectively, and the peak potential difference (Δ*E*_p_) between the two is 266 and 137 mV, respectively. A large Δ*E*_p_ is the prerequisite for achieving the separation of the oxidation peaks of the three substances, which is attributed to the excellent catalytic capacity of GN and the large specific surface area of 3D GN. In addition, the peak current corresponding to the oxidation peak on the GNSs/CC electrodes is larger than that on the CC electrodes, which indicates that GN has high electron transfer ability and can improve the response of the electrode to substances with the same concentration.

**FIGURE 4:**
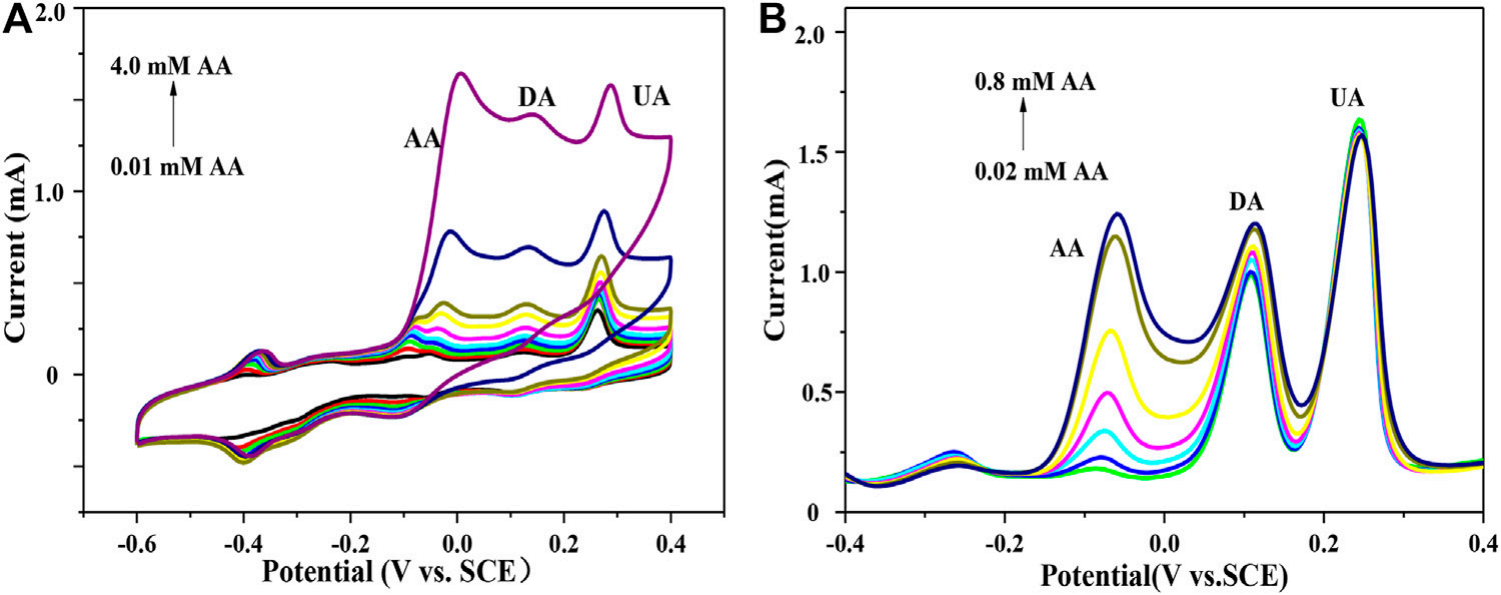
7 h GNSs/CC electrode detects AA in the presence of DA and UA. **(A)** CV. **(B)** DPV.

The GNSs/CC electrodes can distinguish the redox peaks of these three small molecules by CV, detecting them simultaneously. However, CV often presents large non-Faradaic current, which often leads to the extreme drift of peak current (Figure 4A). By contrast, DPV can reduce the limit of voltammetry measurement by significantly reducing the ratio of non-Faradaic current to Faradaic current, thus showing a low detection limit and high sensitivity. As shown in Figure 4B, the oxidation potentials of the three substances detected by DPV are -60 mV (AA), 116 mV (DA), and 244 mV (UA). The Δ*E*_p_ between the two pairs is very large, 176 and 128 mV, respectively. Therefore, the GNSs/CC electrode can meet the requirements of individual and simultaneous detection of these three small molecules. Next, we use DPV to detect the electrochemical properties of electrode materials.

### Differential Pulse Voltammetry for the Detection of Three Small Biomolecules

**FIGURE 5:**
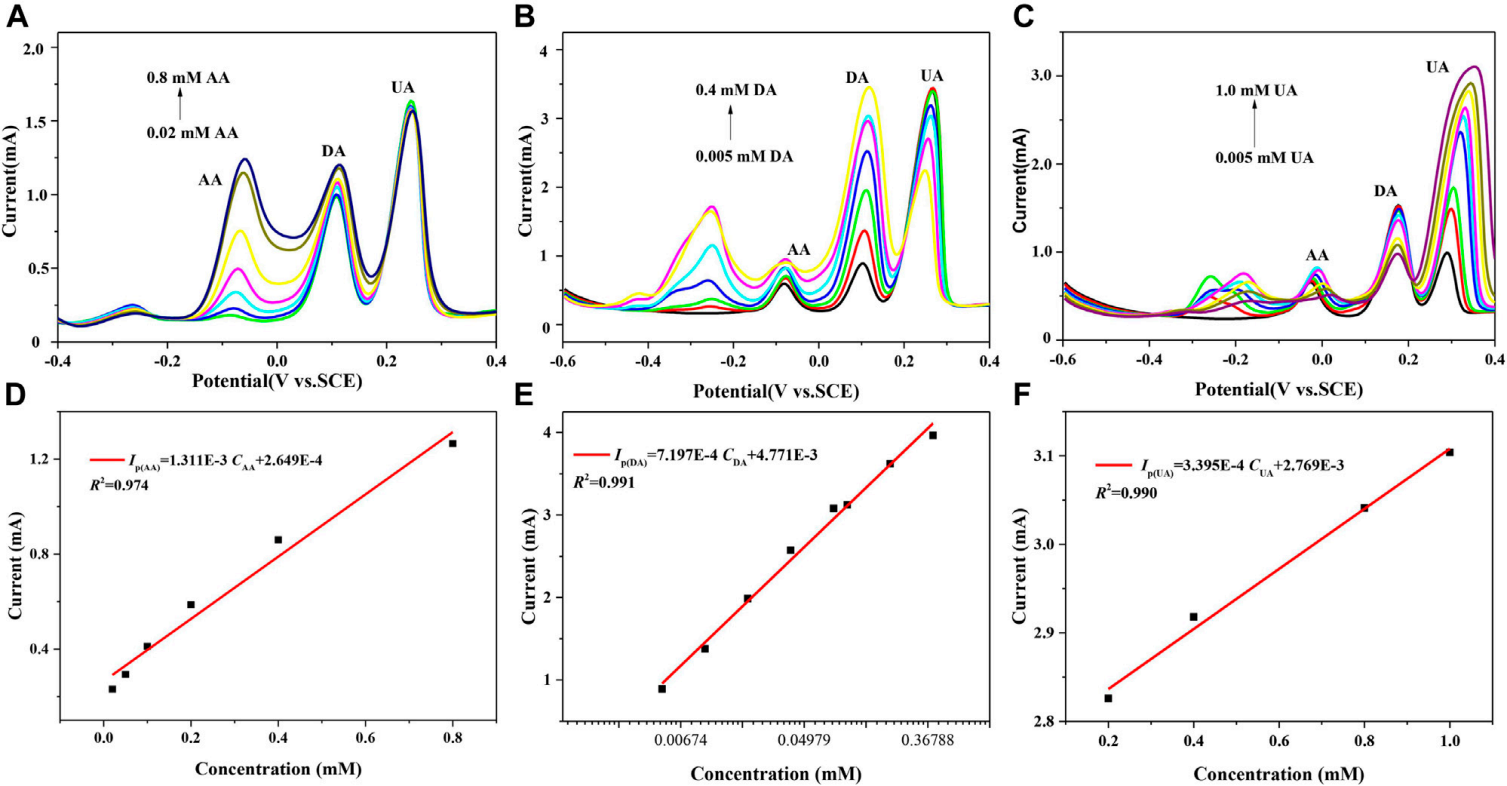
7 h GNSs/CC electrodes are used to detect AA, DA, and UA by DPV. **(A)** AA concentration changes, whereas DA and UA concentrations remain unchanged. **(B)** DA concentration changes, whereas AA and UA concentrations remain unchanged. **(C)** UA concentration changes, whereas AA and DA concentrations remain unchanged. **(D)** The fitting curve of peak current and concentration of AA. **(E)** The fitting curve of peak current and concentration of DA. **(F)** The fitting curve of peak current and concentration of UA.

Figure 5A is the pulsed Volt–Ampere characteristic curves obtained by the simultaneous detection of AA, DA, and UA using 7 h GNSs/CC electrodes by DPV. Figure 5D shows the fitting result of the relationship between the peak current and concentration of AA. In the three mixtures, the concentration of AA varies from 0.02 to 0.8 mM, while the concentrations of DA and UA remain unchanged, which are 0.01 and 0.1 mM, respectively. The results show that the oxidation peak potential of AA is around −60 mV, that of DA is around 116 mV, and that of UA is around 244 mV. There is a large distance between the oxidation peaks of these three substances, and they are completely undisturbed. The peak current of AA increases linearly with an increase in its concentration, while the corresponding peak current of DA and UA almost remain unchanged. In the presence of high concentrations of UA and DA, the sensitivity of 7 h GNSs/CC electrodes for AA detection is 655.5 μA mM^−1^ cm^−2^ and the linear equation is *I*_P_(μA) = 1311C_AA_ + 264.9 (*R* ^2^ = 0.974). Figures 5B,E show the experimental results of detecting DA using 7 h GNSs/CC electrodes by DPV under the condition of constant AA and UA concentrations. The concentration of DA varies in the range of 0.05–0.4 mM, the concentration of AA is 0.1 mM, and the concentration of UA is 0.1 mM. The results show that the oxidation peak potentials of DA, AA, and UA are 116, −76, and 268 mV, respectively. The distance between the oxidation peaks of these three substances is larger, and there is no interference between them. The peak current of DA gradually increases with an increase in its concentration, while the corresponding peak currents of AA and UA almost remain unchanged. In addition, the sensitivity of 7 h GNSs/CC electrodes to detect DA is 359.9 μA mM^−1^ cm^−2^ and the linear equation is *I*_P_(μA) = 719.7C_DA_ + 4,711 (*R* ^2^ = 0.991). Figure 5C shows the pulsed Volt–Ampere characteristic curves of UA detected by DPV using 7 h GNSs/CC electrodes under the condition of constant AA and DA concentrations. The concentration of UA ranges from 0.2 to 1.0 mM, with 0.1 mM for AA and 0.01 mM for DA. The results show that the oxidation peaks of these three substances are also spaced far apart and are not disturbed by each other at all. As UA concentration gradually increases, its peak current also increases linearly between 0.2 and 1.0 mM (Figure 5F). The sensitivity of 7 h GNSs/CC electrodes while detecting UA in the presence of high concentrations of AA and DA is 169.75 μA mM^−1^ cm^−2^, and the linear equation is *I*_P_(μA) = 339.5C_UA_ + 2,769 (*R* ^2^ = 0.990).

**FIGURE 6:**
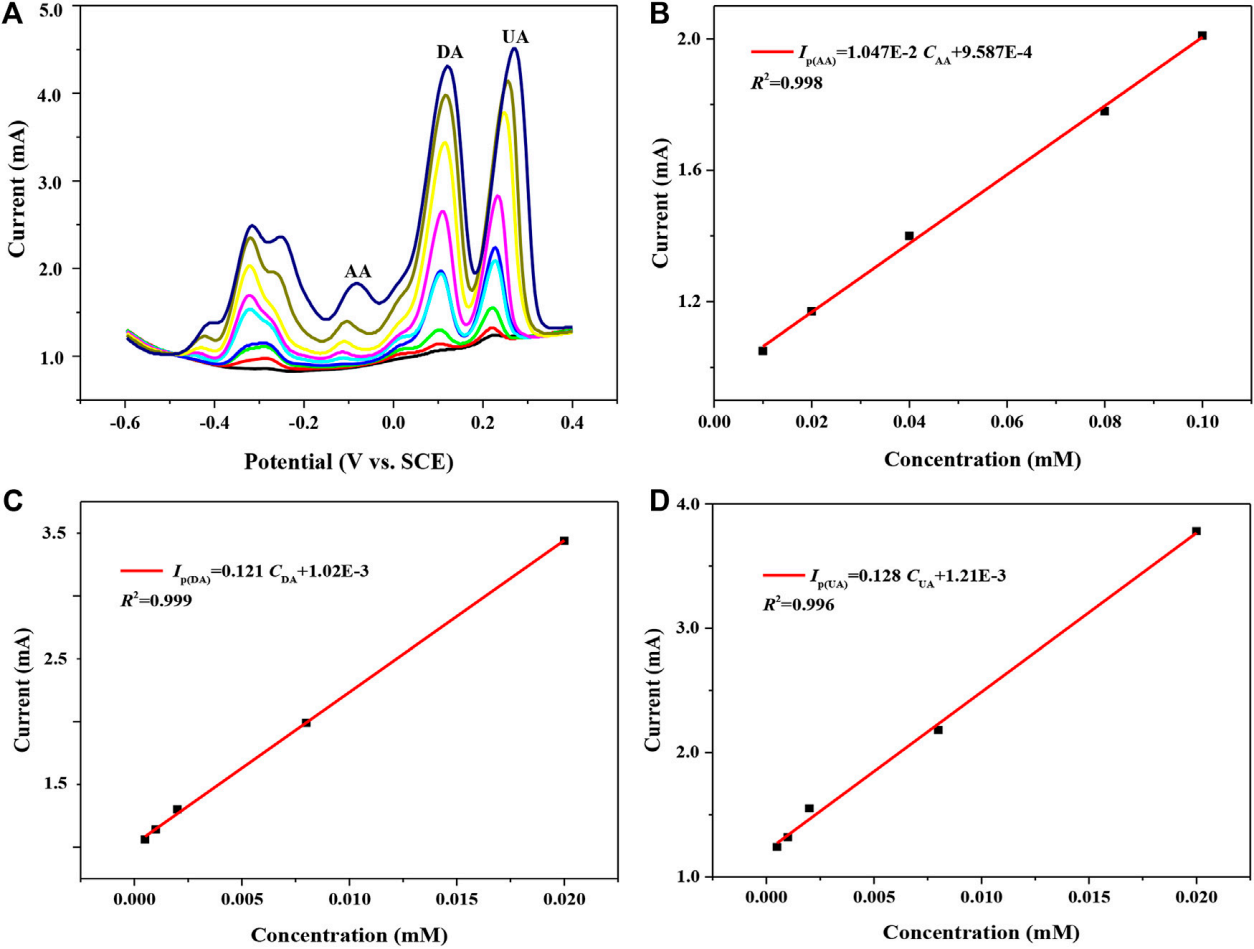
7 h GNSs/CC electrodes are used to detect AA, DA, and UA by DPV simultaneously. **(A)** Pulsed Volt–Ampere characteristic curves of AA, DA, and UA. **(B)** The fitting curve of peak current and concentration of AA. **(C)** The fitting curve of peak current and concentration of DA. **(D)** The fitting curve of peak current and concentration of UA.

Figure 6A shows the pulsed Volt–Ampere characteristic curves of 7 h GNSs/CC electrodes in PBS solution containing AA (concentration is 0.02–0.1 mM), DA (0.0005–0.02 mM), and UA (0.0005–0.02 mM) from −0.6 to 0.4 V potential at a rate of 50 mV s^−1^. The redox process of these three substances can be simply summarized as follows when they are simultaneously detected using 7 h GNSs/CC electrodes: the three small molecules synchronously diffuse to the nearest electrode surface through mass transfer due to the existence of concentration difference, the small molecules diffused to the electrode surface are electrocatalyzed to undergo a redox reaction, and the oxidation products diffuse away from the electrode surface due to the concentration difference. During this process, the 3D-structured GN can not only add the chemical active sites for the oxidation reaction but also serve as an electrolyte reservoir to store more small molecules and improve the electron transfer rate. As shown in Figure 6A, the oxidation peak potentials of AA, DA, and UA are about -84, 120, and 272 mV, respectively. The oxidation peak potential differences of AA and DA and DA and UA are 204 and 152 mV, respectively, indicating that they will not interfere with each other. Moreover, the peak currents of these three substances also gradually increase with the increase in their concentrations. Figures 6B–D show the fitting results of peak currents versus concentration for AA, DA, and UA, respectively. The peak current of AA in the three mixtures is proportional to the concentration in the range of 0.02–0.1 mM, giving a detection sensitivity of 5,470 μA mM^−1^ cm^−2^, and the linear equation is *I*_P_(μA) = 10470C_AA_ + 95,870 (*R* ^2^ = 0.998). The peak current of DA is proportional to the concentration over the range of 0.0005–0.02 mM, with a detection sensitivity of 60,500 μA mM^−1^ cm^−2^, and the linear equation is *I*_P_(μA) = 121000C_DA_ + 1,020 (*R* ^2^ = 0.999). The peak current of UA is proportional to the concentration in the range of 0.0005–0.02 mM, with a detection sensitivity of 64,000 μA mM^−1^ cm^−2^, and the linear equation is *I*_P_(μA) = 128000C_UA_ + 1,210 (*R* ^2^ = 0.996). Compared with the single detection of small molecules, the 7 h GNSs/CC electrodes can obtain ultrahigh sensitivity in the low-concentration region when detecting AA, DA, and UA simultaneously. Additionally, the electron transfer of these three small molecules has a synergistic effect during simultaneous detection.

### Detection of Stability and Anti-Interference

**FIGURE 7:**
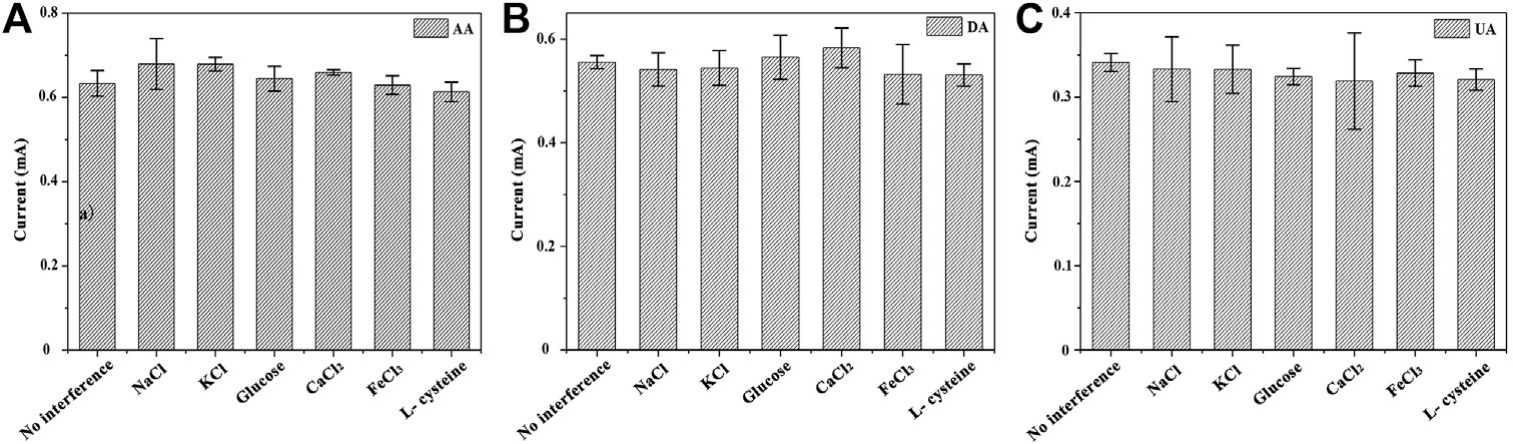
Test results of electrode anti-interference performance of 7 h GNSs/CC electrodes in PBS buffer with pH 7.4. (A) Influence of interfering substances on AA; (B) Influence of interfering substances on DA; (C) Influence of interfering substances on UA.

Research on anti-interference: the anti-interference capability of the GNSs/CC electrodes is very important since the environment of the blood sample is much more complex than the standard solution we prepared. In order to further detect real blood samples and investigate the anti-interference and selectivity of the sensor, we carried out the anti-interference experiment. Some common ions and small molecules coexisting in blood are selected as the research subjects, and the possible ions and molecules are as follows: Na^+^, K^+^, Ca^2+^, Cl^−^, L-cysteine, and glucose. We use 7 h GNSs/CC electrodes to detect the solution (5 mM) containing the above ions and molecules. When three substances (50 μM AA, 5 μM UA, and 5 μM DA) are detected at a certain concentration in the PBS solution at pH 7.4, we judge the effect of the interfering substance on the detection target analyte by recording the current in the presence and absence of the interfering substance (Figure 7). The results show that the current deviation of KCl and CaCl_2_ is relatively larger, which has a certain influence on AA, while the influence of other substances can be basically ignored. NaCl and FeCl_3_ have the greatest influence on DA, while FeCl_3_ and L-cysteine are the most influential substances for UA. On the whole, the common ions and molecules coexisting with AA, DA, and UA have little effect on them, which means that the GNSs/CC biosensor has very good anti-interference ability against potential interfering substances, including ions and small chemical molecules.

Research on repeatability and stability: subsequently, we conducted the repeatability and stability research on the fabricated GNSs/CC sensor. The sensor is sealed in deionized water and stored at room temperature. After 7 days of storage, the same concentrations of AA, DA, and UA are detected again by DPV. The results show that their current values do not change significantly and the responses of AA, DA, and UA are 88.2, 92.5, and 94.2% to the initial current, respectively. In addition, we fabricate three electrodes under the same conditions, on which the three small molecules of AA, DA, and UA are detected by DPV. The results show that the relative deviation of the peak currents of the three small molecules is 2.23% (AA), 2.34% (DA), and 1.35% (UA). The above results demonstrate that the GNSs/CC sensor has good repeatability and stability.

### Detection of Uric Acid in Human Serum Samples

Based on the above discussion, we further apply the GNSs/CC sensor in the detection of real blood samples to explore its practicality. A serum sample with known UA concentration is added to the PBS solution, and the peak current of DPV response is measured. The obtained current value is brought into the fitting relationship between the current and concentration obtained in Supplementary Figure S2, *I*_p_(μA) = 3750 C_UA_ + 763.6, to calculate the concentration of UA in the serum samples. Then, the concentration of UA in raw serum is calculated according to the volume of the dropwise-added serum. Table 1 shows the detection results of UA in the human serum with our GNSs/CC sensor and measurements obtained at Shenzhen Hospital of Peking University. The recovery rate is between 104 and 124% compared with the results measured by dry chemical analysis in the hospital, indicating that the GNSs/CC flexible sensor has good recovery and accuracy in the detection of UA in real blood samples.

**TABLE 1:** Detection of UA in real blood samples.

Serum sample	Results from commercial measurements (mM)	Results of our sensor measurements (mM)	Recovery (%)
1	0.723	0.750	104
2	0.497	0.521	105
3	0.203	0.251	124

Table 2 lists six GN-based sensors and their detection performance. It can be seen that GN and its derivatives are very suitable as the substrate materials for sensors since they can prove their high catalytic performance and excellent electrochemical activity. They can provide similar detection and boosting performance after functional material doping. Compared with all of those sensors, our GNSs/CC sensor offers a good linear range (AA: 20–1,000 μM, DA: 0.5–20 μM, and UA: 0.5–20 μM) and low detection limit (AA: 0.31 μM, DA: 0.01 μM, and UA: 0.03 μM; S/N = 3) without further functional material doping. The sensor still holds remarkable potential in performance boosting of biomedical detection. In addition, our sensor is fabricated on the substrate of CC, which can be integrated with flexible electronics to fit many biomedical detection and sensoring scenarios.

**TABLE 2:** Performance comparison of sensors for small molecule detection.

Sensor	Linear range (μM)			Detection limit (μM)			References
	AA	DA	UA	AA	DA	UA	
PrGO/MnO_2_	1–800	0.03–45	0.3–80	1.00	0.02	0.05	Tukimin et al. (2018)
Pd_3_Pt_1_/PDDA-RGO	40–1,200	4–200	4–400	0.61	0.04	0.10	Yan et al. (2013)
Au@Pd-RGO	1–800	0.1–100	0.1–350	0.28	0.024	0.02	Jiang and Du, (2014)
rGO-CNT/ITO	10–200	0.2–8.0	0.2–16.0	5.31	0.04	0.17	Zhang et al. (2015)
AuNPs/P(PDA)/GO	6.0–2,400	0.05–100	0.5–150	1.76	0.017	0.16	Tig et al. (2017)
ErGO/CFE	8–2016.45	1.5–224.82	6–899.3	4.5	0.77	2.23	Yang et al. (2014)
GNSs/CC	20–1,000	0.5–20	0.5–20	0.31	0.01	0.03	Our work

## Conclusion

In summary, we have successfully fabricated a GNSs/CC flexible sensor via thermal CVD that can detect AA, DA, and UA simultaneously. The GNSs/CC electrodes show good electrocatalytic activity in the detection of DA, AA, and UA, and the optimized performance is obtained when the GN growth time is 7 h. The ultrasensitive simultaneous detection of these three small molecules is realized by DPV using 7 h GNSs/CC electrodes, which can reduce the non-Faradaic current drift. The working linear range and sensitivity of the sensor are obtained. The linear ranges for the detection of AA, DA, and UA are 0.02–0.1, 0.0005–0.02, and 0.0005–0.02 mM, respectively, and their sensitivity rates are 5,470, 60,500, and 64,000 μA mM^−1^ cm^−2^, respectively. The flexible sensor has good performance in terms of reproducibility, stability, and anti-interference against common interfering substances in the blood. Moreover, it has successfully detected UA in the human serum with the recovery rate between 104 and 124%, indicating that the GNSs/CC sensor holds remarkable potential in the commercial detection of real biomedical samples. This work provides an ultrasensitive detection tool and method for the simultaneous detection of the basic substances AA, DA, and UA that sustain the metabolic activities of living organisms.

## Data Availability

The original contributions presented in the study are included in the article/Supplementary Material; further inquiries can be directed to the corresponding author.
