# Supplementary material for: Graphene-Based Flexible Sensors for Simultaneous Detection of Ascorbic Acid, Dopamine, and Uric Acid
Source: Front Bioeng Biotechnol. 2021 Sep 20;9:726071. doi: 10.3389/fbioe.2021.726071 (PMC8488115; doi:10.3389/fbioe.2021.726071)
Supplement: Supplementary file 1 [file DataSheet1.docx]

Supplementary Material


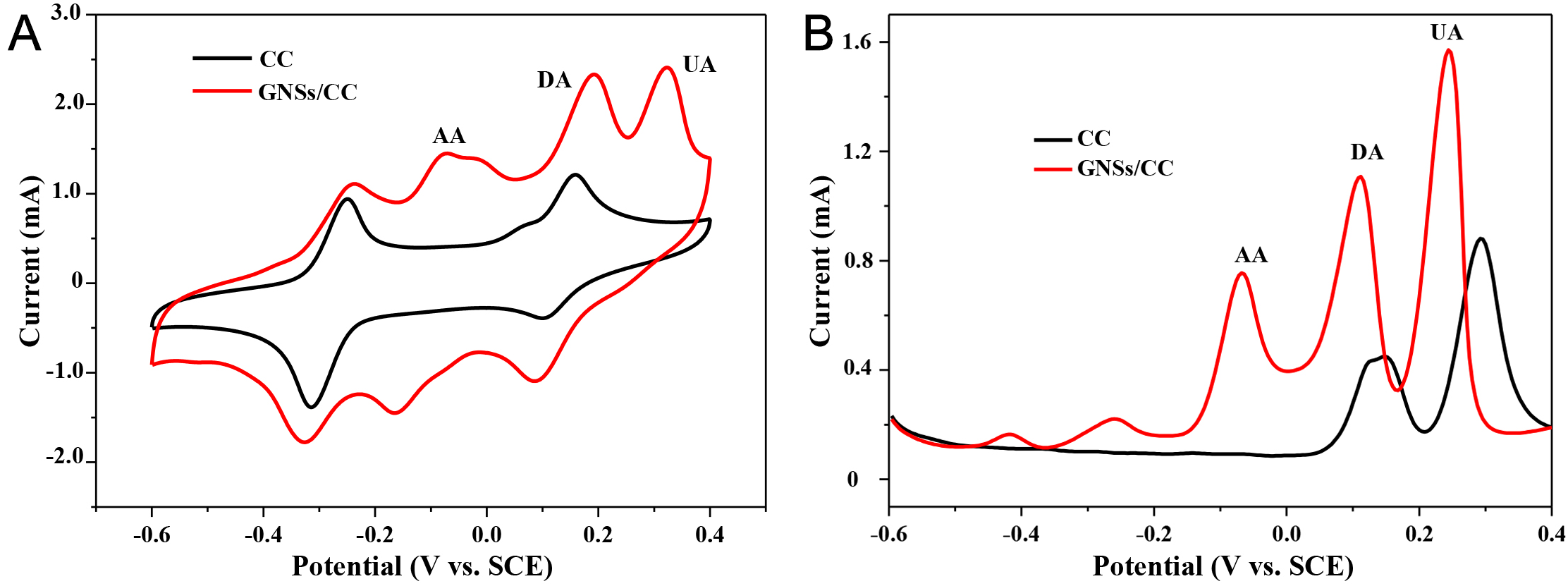


**Supplementary Figure 1.** CC and GNSs/CC electrodes are used to simultaneously detect AA, DA, and UA, respectively. (A) CV; (B) DPV.

Supplementary Figure 2A shows the differential pulsed volt-ampage characteristic curves obtained by measuring different concentrations of AA from -0.6 to 0.4 V at a rate of 50 mV s^-1^ using the 7 h GNSs/CC electrode with the best comprehensive performance in 0.1 M PBS solution. The results show that the oxidation peak potential of AA is -96 mV, and when the concentration of AA increases, its oxidation peak potential hardly moves. Supplementary Figure 2D is the fitting result of the corresponding current and concentration. As the concentration of AA increases, the corresponding peak current gradually increases and the peak current is linearly correlated with the corresponding concentration. The linear equation is: *I*_P_(μA) = 3399C_AA_ + 750.2 (R^2^ = 0.989). The linear concentration range is 0.005 to 0.4 mM, the detection sensitivity is 1700 μA mM^-1^ cm^-2^, and the minimum detection concentration is 0.001 mM. Then we detect DA and UA with CV respectively. Supplementary Figures 2B,E show that the oxidation peak potential of DA is 124 mV, and the potential hardly moves when the concentration of DA increases. As the concentration of DA increases, the corresponding peak current gradually increases and the peak current is linearly related to the corresponding concentration. The linear equation is: *I*_P_(μA) = 310C_DA_ + 2845 (R^2^ = 0.990). The linear concentration range is 0.005 to 1.0 mM, the detection sensitivity for DA is 155 μA mM^-1^ cm^-2^, and the minimum detection concentration is 0.0001 mM. As shown in Supplementary Figures 2C,F, the oxidation peak potential of UA is 264 mV, and when the concentration of UA increases, the position of the oxidation peak potential almost does not move. With the increase of UA concentration, the corresponding peak current gradually increases and the peak current is linearly related to the corresponding concentration, and the linear equation is: *I*_P_(μA) = 3750C_UA_ + 763.6 (R^2^ = 0.994). In addition, the linear concentration range is 0.004 to 0.1 mM, the sensitivity for UA is 1875 μA mM^-1^ cm^-2^, and the minimum detected concentration is 0.001 mM.


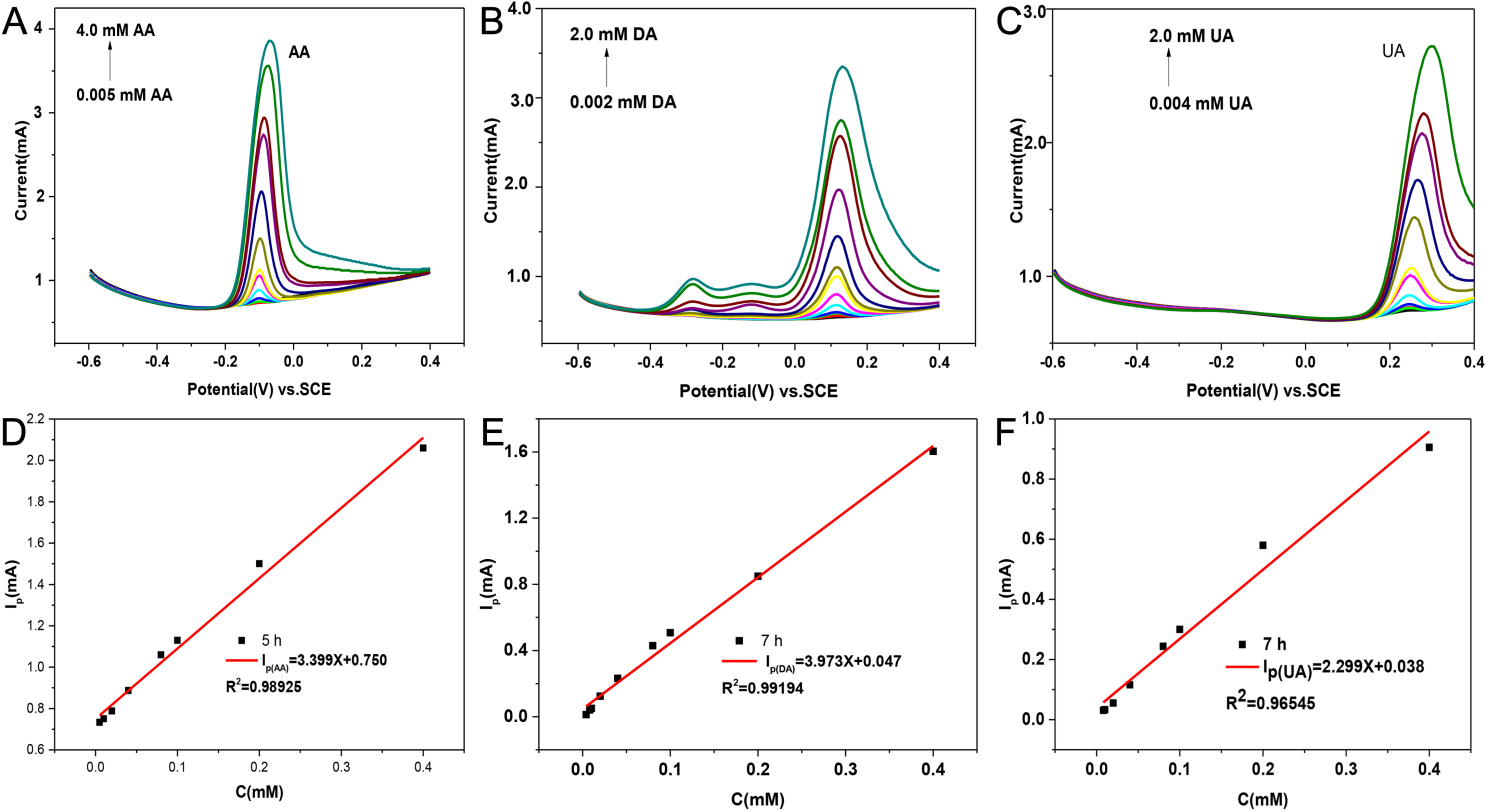


**Supplementary Figure 2.** 7 h GNSs/CC electrodes are used to separately detect AA, DA, and UA by CV. (A) CV to detect AA alone; (B) CV to detect DA alone; (C) CV to detect UA alone; (D) Fitting result of the relationship between current and concentration is obtained by detecting AA using 7 h GNSs/CC; (E) Fitting result of the relationship between current and concentration is obtained by detecting DA using 7 h GNSs/CC; (F) Fitting result of the relationship between current and concentration is obtained by detecting UA using 7 h GNSs/CC.
